# Development of a bowel sound detector adapted to demonstrate the effect of food intake

**DOI:** 10.1186/s12938-021-00969-2

**Published:** 2022-01-04

**Authors:** Ning Wang, Alison Testa, Barry J. Marshall

**Affiliations:** 1 The Marshall Centre for Infectious Diseases Research and Training, University of Western Australia, Perth, 6009 Australia; 2 Noisy Guts Pty Ltd, Level 2, L-block, QEII Medical Site, Nedlands, WA 6009 Australia

**Keywords:** Bowel sounds, CNN, Food intake

## Abstract

**Objective:**

Bowel sounds (BS) carry useful information about gastrointestinal condition and feeding status. Interest in computerized bowel sound-based analysis has grown recently and techniques have evolved rapidly. An important first step for these analyses is to extract BS segments, whilst neglecting silent periods. The purpose of this study was to develop a convolutional neural network-based BS detector able to detect all types of BS with accurate time stamps, and to investigate the effect of food consumption on some acoustic features of BS with the proposed detector.

**Results:**

Audio recordings from 40 volunteers were collected and a BS dataset consisting of 6700 manually labelled segments was generated for training and testing the proposed BS detector. The detector attained 91.06% and 90.78% accuracy for the validation dataset and across-subject test dataset, respectively, with a well-balanced sensitivity and specificity. The detection rates evaluated on different BS types were also satisfactory. Four acoustic features were evaluated to investigate the food effect. The total duration and spectral bandwidth of BS showed significant differences before and after food consumption, while no significant difference was observed in mean-crossing rate values.

**Conclusion:**

We demonstrated that the proposed BS detector is effective in detecting all types of BS, and providing an accurate time stamp for each BS. The characteristics of BS types and the effect on detection accuracy is discussed. The proposed detector could have clinical application for post-operative ileus prognosis, and monitoring of food intake.

## Background

Bowel sounds (BS) or gut noises are the rumbling, gurgling or whining noises, produced by the movement of food, liquids and gases through the intestine. Bowel sounds can be used in the prognosis and diagnosis of gastrointestinal conditions. For example, doctors have listened to gut noises with stethoscopes for centuries, and used them to diagnose conditions. The location, intensity and pitch of the sounds are all considered clinically important [1]. High pitched sounds can indicate a bowel obstruction and absence of bowel sounds after surgery can indicate an ileus (lack of propulsive movement) before the patient starts vomiting or complaining of abdominal pain.

Recent increases in computer processing power and improvements in data analysis bring the potential to employ bowel sound computational analysis to improve evaluation of gastrointestinal conditions [2] or feeding status [3, 4]. A number of computerized bowel sound-based monitoring and diagnosis systems have been developed [5, 6]. The development stage of the various technologies varies. The most advanced appears to be the AbStats® device from GI Logic, which is registered for the USA market and can be used in the prognosis of post-operative ileus [7, 8]. Other research groups have also focused on this important clinical problem [9]. Accurately predicting whether a patient will suffer from ileus after abdominal surgery can prevent complications in those affected, and allows unaffected patients to move through the post-operative feeding stages quickly, freeing up space in hospitals.

Monitoring BS may also be useful as an objective method to track food intake. Cohen et al. [10] demonstrated proof of concept for use of the AbStats® device to track intestinal rate and hence discriminate between ingestion of a large, high calorie meal and a small, low calorie meal, through changes in the rate of bowel sounds at 90 min and 270 min post-ingestion. This paved the way for use of BS in development of a weight loss tool that enables physiologically based ingestion guidance to complement traditional obesity management.

Since BS occur in an irregular pattern, an important first step for any BS-based diagnosis or monitoring system is to extract the BS segments of the recording, while neglecting those segments that only contain background noise. Improved accuracy at this stage will improve to the accuracy of any subsequent signal processing or analysis. A recent overview of automatic bowel sound analysis methods, also suggested that the detection of the BS occurrence could be a starting point for building bowel sound analysis standards, as well as international forums for discussion and cooperation [6].

Early researchers in this space employed statistic-based methods to extract BS [11, 12]. Rekanos and Hadjileontiadis proposed an iterative kurtosis-based technique for the detection of bowel sound in 2006 [11], and Ulusar developed a gastrointestinal motility monitoring system using a Naive Bayesian algorithm for BS observation in 2014 [12]. Subsequently, several advanced machine learning techniques were employed for BS detection, including support vector machine (SVM) [13], artificial neural network (ANN) [14, 15], and long short term memory (LSTM) [16] approaches, and have achieved better results than the traditional methods. However, even these advanced models have some limitations. The classic machine learning models coupled with simple hand-crafted features [13, 15], become less effective when the sound recordings are contaminated by different types of noise [17]. On the other hand, models with more parameters, such as LSTM with time domain sequence as input, are prone to over-fitting. This is due to the great variation in the BS length and the time interval between BS, and the weak temporal correlation of the adjoining BS events [17].

Most recently, use of the convolutional neural network (CNN) method, has been introduced in this area [17, 18]. Preliminary results for this method are promising, with high accuracy and sensitivity. However, in the published studies to date, the CNN models were limited to identification of the presence of bowel sounds in one second or longer segments. This may be problematic since of all the types of BS, short BS of approximately 20–40 ms duration (variously described as single bursts, solitary clicks or intestinal bursts), are the most common type [19]. Hence, we assume that a system analysing a shorter segment window would be better for identifying the majority of BS. In addition, the acoustic features obtained from BS, such as the duration and energy, have found to be useful for evaluating the bowel motility [12, 20, 21]. To accurately calculate these features, the start and end time point of each BS are required. These are not provided by the documented CNN models.

We here describe an improved CNN-based BS detector that is able to extract both short and long BS. The structure of the CNN is designed to work on narrow inputs, and hence is able to provide accurate predictions on thinly sliced audio recordings and provide information about start and end points. We used the Mel spectrum as input to convert the one-dimensional sound segment into a two-dimensional spectrogram. BS were recorded in 40 healthy volunteers before and after food intake and were manually labelled and used to form a large dataset. Experimental results show that the CNN-based BS recognizer achieves over 90% accuracy and is high in both sensitivity and specificity.

The BS detection results were then used to investigate the effect of the food on sound. We extracted four acoustic features from BS: the total duration of BS per minute, spectral centroid (SC), spectral bandwidth (SBW), and mean-crossing ratio (MCR). The findings may help drive development of more accurate tools to track food intake as part of obesity management.

## Results

### Collected audio recordings and generated BS dataset

Audio data from 40 participants were collected and labelled for building a BS dataset. Two sensors were placed on the participant abdomen, one on the right lower quadrant (RLQ) and another on the left upper quadrant (LUQ). A stretchy belt is used to apply a small tension on the sensor to keep it firmly at the right place, without being excessively tight nor changes the gastrointestinal condition. A 2-h-long recording during fasting and a 40-min recording after food were collected from each study participant. Eighty recordings, with a total length of over 100 h, were therefore collected. Random segments were cut from audio files and manually labelled by human experts. The start and end point of BS segments were then carefully adjusted to make sure one BS per segment only. Finally, a total of 6700 audio segments were labelled, including around 500 s of BS segments with each segment ranging from 0.03 s to 1.6 s, and more than 3000 s of non-BS segments with each segment ranging from 0.1 s to 5 s. Thirty-six thousand image samples were generated to form the dataset for developing the CNN model. The samples were split into three parts: a balanced training and validation set, which included 22,400 training and 9600 validation samples from 35 subjects, and a balanced across-participants blind testing set including 4000 BS and non-BS samples from an additional five subjects. The subjects in the across-participants test set were randomly selected from subjects that had more than 100 labels of BS segments, to ensure the size of the test dataset.

**Fig. 1:**
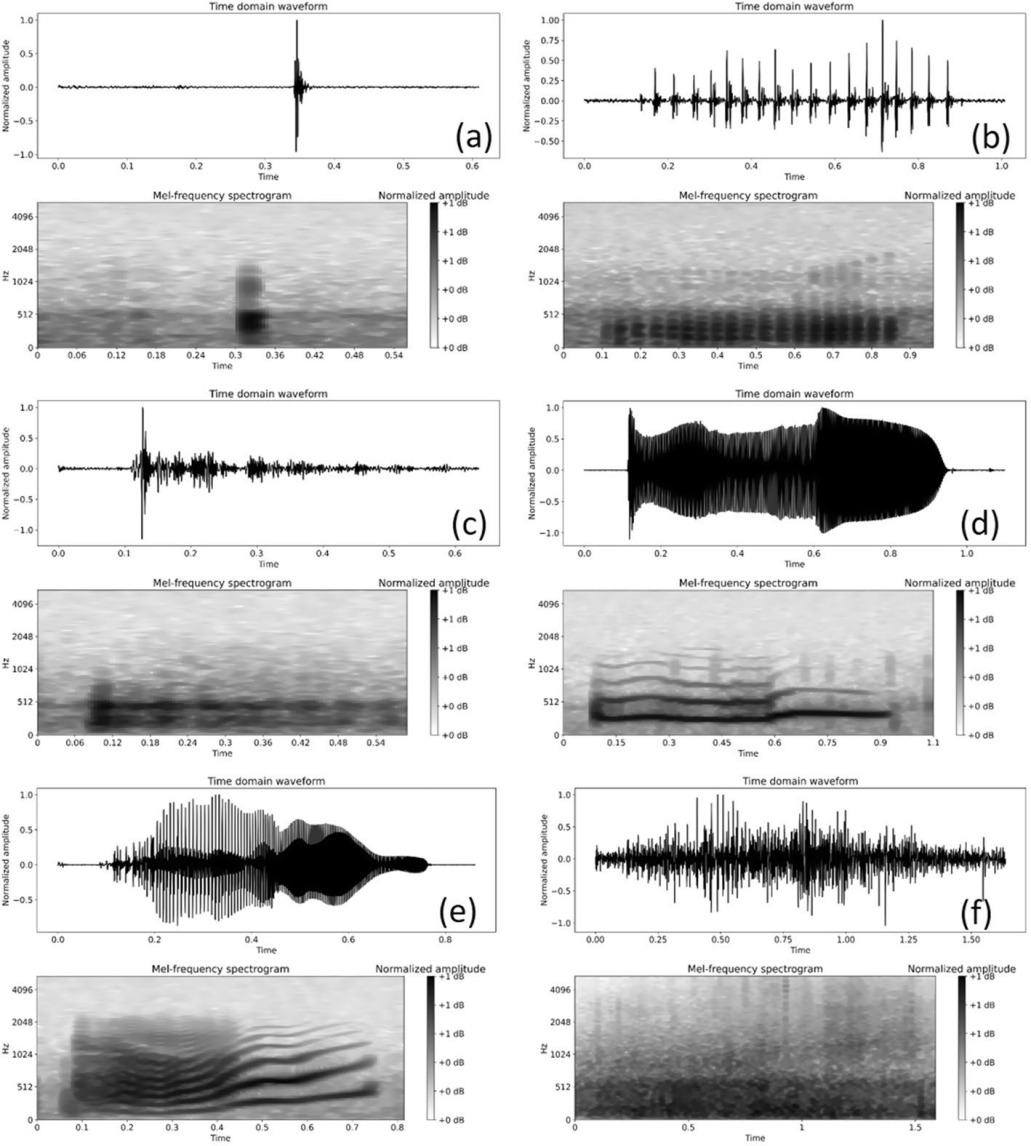
Example of collected BS subtypes in time domain (top) and its spectrogram (bottom). **a** SB; **b** MB; **c** CRS; **d** HS; **e** HS with frequency changing over time; and **f** noise segment (the relative abundance of BS types is: SB > MB > CRS > HS, see Du et al. [19])

Figure 1 shows waveforms and spectrograms of typical noise and bowel sound segments recorded by our data acquisition system. Previous research has shown that BS can be divided into subtypes, including single burst (SB), multiple bursts (MB), continuous random sound (CRS), and harmonic sound (HS), and the characteristics of these subtypes are quite different. The proportion of each BS subtype also varies, as shown in Du et al. [19], from over 80% to less than 1%. It is therefore necessary to test whether the developed detector is able to detect all BS subtypes or only the most common types of BS. 2000 BS samples were selected from the validation set and further labelled by the human experts for this purpose, including 500 samples for each BS subtype.

### Performance of the BS detector

The sensitivity, specificity and accuracy were calculated to evaluate the performance of the proposed CNN classifier. Specifically, sensitivity and specificity are defined as follows:

1 $$ {\text{Sensitivity}} = {\text{TP}}/\left( {{\text{TP}} + {\text{FN}}} \right), $$

2 $$ {\text{Specificity}} = {\text{TN}}/\left( {{\text{TN}} + {\text{FP}}} \right), $$

where the TP are the numbers of the samples derived from BS segments that are correctly identified as BS, and TN are samples derived from non-BS segments that are predicted as noise. FP and FN are the corresponding false predictions. When testing the accuracy of the developed BS detector on the BS types, the accuracy is defined as the proportion of samples from each BS subtype group that are correctly identified as BS.

The results of the detector on the validation set and external across-participants test set are listed in Table 1. The model shows fairly balanced sensitivity and specificity on both datasets, and a high accuracy above 90%. This implies that the generalizability of the proposed model is quite good. The detection accuracy for different BS types is listed in Table 2.

**Table 1:** Performance of BS detector

	**Sensitivity (%)**	**Specificity (%)**	**Accuracy (%)**
Validation dataset	91.28	90.84	91.06
Across participants test set	90.34	91.20	90.78

**Table 2:** Performance of BS detector for different BS subtypes

BS type	Accuracy (%)
SB	99.70
MB	93.59
CRS	78.34
HS	98.77

### Results of food intake on BS acoustic features

**Fig. 2:**
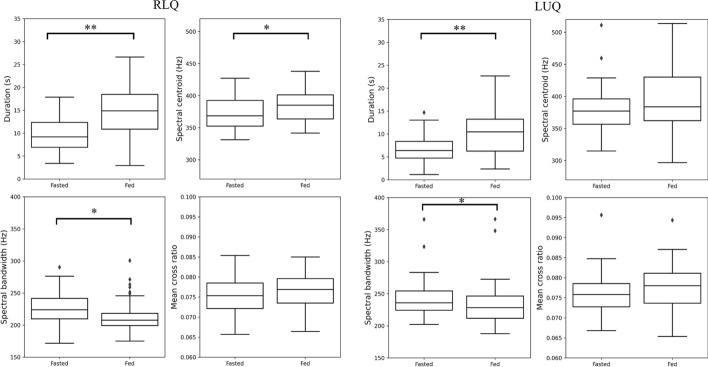
Comparison of differences in BS acoustic features before and after food intake (*: *p* < 0.05, **: *p* < 0.001)

Figure 2 shows the box plots of the BS acoustic features, calculated from all BS recorded in the before and after the food recordings, respectively. As Table 3 shows, significant differences arose in the total duration per minute of BS and spectral bandwidth before and after food recorded from both RLQ and LUQ, and also in the spectral centroid calculated based on BS from RLQ. Generally, the significance level is higher for the BS features from RLQ.

**Table 3:** Characteristics of BS acoustic features: median (interquartile range)

	**RLQ**		**LUQ**	
	**Fasted**	**Fed**	**Fasted**	**Fed**
Duration	9.5 (5.4)	14.9 (7.6)	6.4 (3.7)	10.5 (7.0)
SC	368.6 (40.1)	385.2 (37.5)	377.1(39.6)	383.7(67.8)
SBW	224.0 (31.9)	207.8 (19.0)	235.9(29.9)	228.4(34.7)
MCR	0.075(0.006)	0.077(0.006)	0.075(0.006)	0.078(0.007)

In contrast, no significant difference was observed in mean-crossing ratio at any of the sensor channels.

## Discussion

### Performance of the BS detector

The developed CNN-based BS detector in this study achieved an accuracy of 91.06% and a well-balanced sensitivity and specificity. All four subtypes of BS were successfully extracted by the detector.

Almost all samples of SB and HS subtypes were correctly predicted. The SB is a simple pulse, usually with a clear peak showing up in the spectrogram and no other SB present within 100 ms on either side. HS are whistling-like sounds and have one to a dozen frequency components in the spectrogram. The highest harmonic frequency of a HS recorded in the study was up to 4000 Hz. These characteristics make the spectrograms of these two types of BS spiky, and therefore are easily distinguishable from spectrograms of noise segments, which are usually flat. Therefore, it is not surprising to see the high detection accuracy for these two subtypes of BS. HS may be indicative of an obstruction, making this finding of particular clinical value.

We also demonstrated a high detection rate for MB (93.87%). An MB can be described as a cluster of SB. Each burst in a MB looks quite similar, while the amplitude of the pulse and the frequency bandwidth might have some slight differences. There are clear silent gaps between the adjacent bursts, and the length of these silent gaps are also inconsistent. The slight decrease of the accuracy is possible due to the existence of these silent gaps.

The CRS samples had the lowest accuracy, but this was still around 80%. CRS is a continuous random sound, and has everything clustered together, without clear rhythm or pattern. The lack of clear pattern gives it more variability, and also makes it more difficult to be recognized. However, we believe that the accuracy of detecting CRS could increase with a more comprehensive dataset of CRS samples.

Our detector has some advantages when compared to other methods developed to extract BS. Zhao K et al. [17, 18] developed CNN-based BS recognizers, and found similar accuracy to our study (above 90%) However, their models were designed to be low computational complexity solutions, and therefore were restricted to identify if a bowel sound had appeared in a segment of 1 s duration. They were successful in their aim. However, unlike our detection they do not provide a time stamp for the BS.

Liu J., et al. [16] developed an LSTM-based BS detector, which is able to determine the beginning and ending points of every bowel sound by segmenting the recording and detecting BS on each segment. Although they reported a high accuracy and sensitivity on the test set at above 90%, when they tested the model under ‘the real use’ condition, the sensitivity dropped to 62%.

Horiyama et al. [20] has recently reported an ANN-based BS detector, with improved power-normalized cepstral coefficients. Their model achieved good accuracy under both quiet and noisy environments. The unbalanced dataset used in their work, however may have led to potential problems, because they also reported a low positive predictive value, at around 60% ± 20%.

With our proposed detector we achieved a similar accuracy performance to other researchers, but with both high sensitivity and specificity. Further, our comparatively large study demonstrates the good generalizability of our detector. The method also facilitates the ability to give an accurate start and end point of each BS (see methods section for detail), which is important for calculating the BS features, and for BS feature based analysis. In addition, the accuracy of our model in detecting bowel sound types SB, MB and HS was excellent. The model could be further improved with a more comprehensive dataset for CRS samples. The extra information provided by BS features or the occurrence of number of specific BS subtypes may provide additional clinical insight.

### Effect of food intake

We found a significant increase in total BS duration after food consumption. Similar results have been reported previously and this consistency further validates our approach. Du et al. [21] showed that the BS density and summed amplitude were significantly higher after food consumption. Recently, Horiyama et al. [20] also reported that the density and length of BS significantly increased after coffee and soda intake.

The frequency domain BS features, SC and SBW, show different results. The spectral bandwidth shows significant difference between the two recording periods while the spectral centroid shows significance only at RLQ. The spectral centroid is the “centre of mass” of the spectrum, and the spectral bandwidth is defined as the frequency range in which the amplitude is not less than half its maximum value. The results suggest that, the food intake would shift the main frequency component for BS from RLQ, and reduce the energy spread of the frequency spectrum for BS recorded from both RLQ and LUQ.

The MCR is the number of times the waveform crosses its mean value. Previous research by our research group, as documented in Fig. 3b in Du et al. [19], shows that the distributions of MCR have the most significant difference between BS subtypes. This is because that the non-BS intervals, similar to the unvoiced periods, usually have larger MCR values than BS, which are similar to voiced periods [22]. And the interval time between bursts within a BS is one of the main differences between BS subtypes. Therefore, the very small difference in MCR value between the two periods indicates that food intake likely does not have much influence on the proportion of subtypes among all generated BS.

Moreover, generally the significance level is higher for the BS features from the RLQ. This could be explained by the sensor placement. The sensor located on the RLQ of the participants, is placed near the ileocecal valve, which controls the flow of digested food passing from the small intestine into the large intestine. Therefore, the motion of the valve is largely affected by the food intake, and is reflected in the changes of acoustic features of the generated BS.

## Conclusion

In this study, a method of detecting BS based on the convolutional neural network was developed and shown to be effective. The network was well trained by a large amount of data, generated from recordings from two sensors placed on 40 participants. The model showed an accuracy rate of 91.06%, a well-balanced sensitivity and specificity, and a good generalization ability in the across-subject test set.

In addition, the proposed model is excellent at capturing SB, MB and HS. This demonstrates the practical value of the proposed model: it has previously been reported that SB and MB are the most common types of BS, and HS are indicative of obstruction.

The BS detection results obtained using the developed CNN classifier in this study were then used to investigate the effect of the food intake. Several acoustic features are evaluated and different responses to the food intake were found. The results suggest that meal intake increases the total length of BS, change the main frequency component and also constrain the energy spread over the frequency spectrum.

The proposed BS detector could have multiple clinical applications, most obviously to improve bowel sound detection for post-operative ileus prognosis. We also believe that applying the BS characteristic based method could be useful for monitoring food intake. Although the short BS are the most common type seen in the recordings, the long BS shows a higher potential for distinguishing food intake.

## Method

### Clinic-appropriate bowel sound acquisition system

**Fig. 3:**
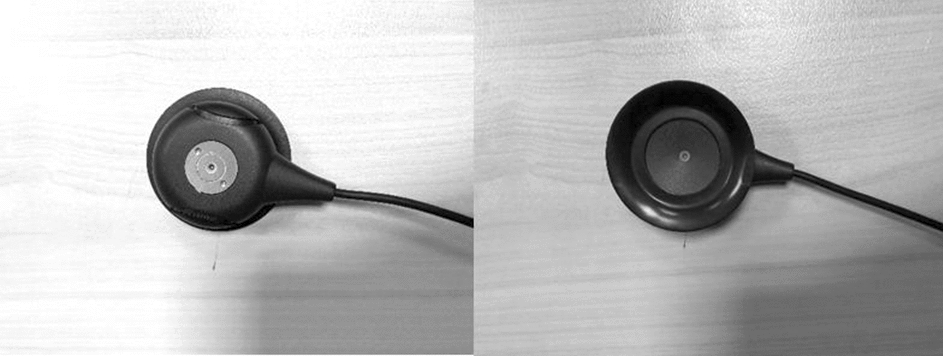
MEMS sensor head

A clinic-appropriate signal acquisition system with Micro-Electro-Mechanical systems (MEMS) microphone-based sensor heads was used in this study. The configuration of a single sensor head is shown in Fig. 3. The sensor head consisted of two microphones. The inward-facing microphone sits close to the abdomen of the subject and predominantly records the BS, while the outward-facing microphone picks up the environmental noise. The design reflects the intention to later develop active noise reduction as part of signal processing allowing for optimal use of the technology in noisy clinical settings. However, for this study, recordings were made in a quiet setting, and only the recordings from the inwards facing microphone were used.

The sensor heads were connected to a central recording unit via cables. The signals acquired by the microphones were transformed to 16-bit PCM data with a sampling frequency at 11 kHz. The recording unit stores the audio data to a removable SD card, allowing subsequent transfer of the data to a desktop for analysis.

**Fig. 4:**
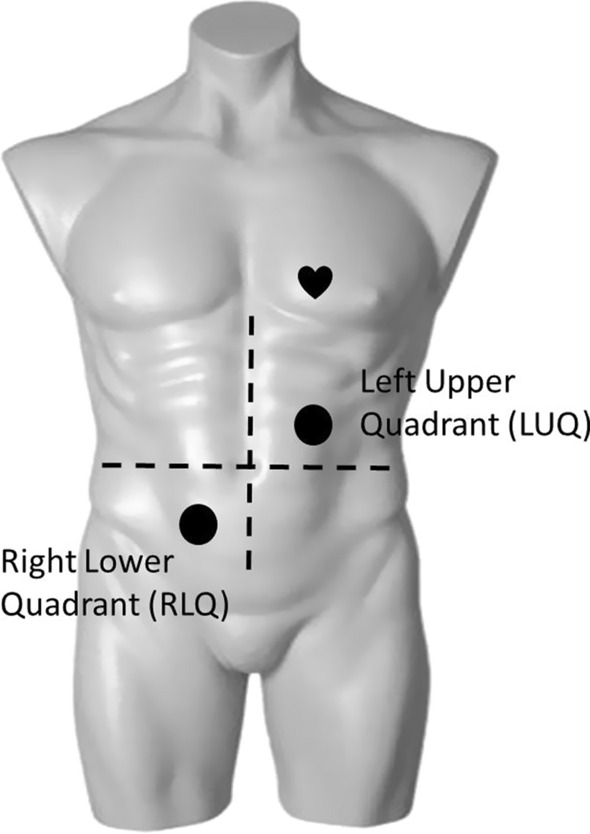
Sensor placement on human body

Figure 4 shows the sensor placement on the participant’s body. The sensor on RLQ records BS from small and large intestine, while the sensor placed on LUQ is mainly recording BS from stomach and transverse colon.

### Data collection

The audio data were collected in a non-clinical setting at the Marshall Centre on the QEII Medical Centre site. The BS recordings of 40 participants were collected for building the bowel sound dataset. All participants were aged between 18 to 65 years old, and had a body mass index (BMI) above 18.5. The study was conducted in accordance with ICH Good Clinical Practice guidelines and approved by The University of Western Australia’s Human Research Ethics Committee (Study number RA 4/1/8893).

Each study participant fasted overnight prior to the first recording, they then consumed a standard meal of a Sustagen® shake and small glass of water prior to completing a second recording. The recordings were collected under quiet conditions, and the participants were asked to sit quietly in armchairs and had access to the internet and reading material. After the recordings were collected, segments were randomly cut from audio files, and manually labelled by one of four individuals experienced in identifying bowel sounds of all types as BS and non-BS segments using Audacity® [23]. If the first labeller was unsure of the label, segments were cross checked by the most experienced member of the team. Since the length of BS could have a large variation, from tens of millisecond to several seconds, for a long BS segment, multiple 60 ms samples were sliced to ensure the uniform size of the input data for the CNN model.

### CNN-based BS detector

The BS detector comprised the following steps: firstly, the sound was filtered by a second-order Butterworth high-pass filter with a cut-off frequency of 80 Hz. Then the normalized Mel-scaled spectrograms were calculated from each of the labelled samples, with frame length of 50 ms, and frame shift of 5 ms. Hence, for each signal piece, the shape of the input tensor was 128*3.

**Fig. 5:**
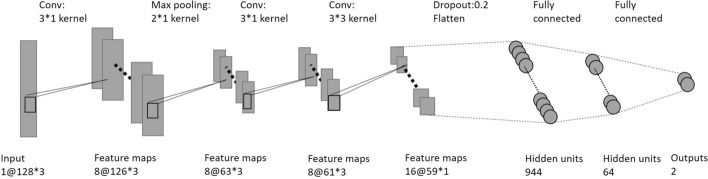
The specific structure of the CNN classifier used in this work

A typical structure of CNN classifier is composed of three components: convolutional layers, pooling layers, and fully connected layers. The short duration of BS limited the structure of our network (Fig. 5), because the width of the input image was only 3 pixels. After three convolutional layers with the rectified linear units activation and max pooling layers, a drop out layer equal to 0.2 was used, to prevent over-fitting. The output was then flattened and sent to a 64 dense layer. A fully connected softmax layer was used to get the BS or non-BS prediction for the input sample.

For the training phase in this experiment, the Adadelta solver was used for weight updating. The model was trained for 30 epochs with the batch size set as 128. Every time an epoch was completed, the accuracy of the model on the validation set was computed. After training finished, the model was tested on the external test set. The recognizer was built using TensorFlow (version 2.1.0) package [24] in Python, and was trained on a GeForce RTX 2080 GPU. The wavio [25] package was used to read the audio files and librosa (version 0.8.0) package [26] was used for generating the input spectrograms.

The BS detector would be used to identify BS on an audio recording. The Mel-scaled spectrograms of the full recording were calculated through a moving window, with a hop length of 50 ms. These spectrograms would be fed into the BS detector in time order, and the detector would produce a time series of predictions (BS or non-BS). Segments containing BS are therefore identified where at least three adjacent spectrograms were predicted as BS. The start time for a BS is defined as the time point of the first occurrence of a BS prediction after non-BS periods, and the end time is defined as the start point of the following non-BS predictions. Based on previous research [19], BSs present within 100 ms around each other were merged as one BS, and the non-BS periods between these BSs were seen as intervals between bursts.

### Effect of food intake

We evaluated the effect of food intake on the characteristics of BS using the developed BS detector. Four typical acoustic features including the total duration of BS per minute, SC, SBW, and MCR, before and after food intake, were investigated. The Wilcoxon test was used to compare the features before and after food intake. A total number of 8 comparisons were made, and the p values were adjusted based on Bonferroni correction method [27] to avoid the issue of multiple testing. The levels of *p* < 0.05 and *p* < 0.01 were reported.

## Data Availability

The datasets used and/or analysed during the current study are available from the corresponding author on reasonable request.

## Abbreviations

BS: Bowel sounds
SB: Single burst
MB: Multiple bursts
CRS: Continuous random sound
HS: Harmonic sound
BMI: Body mass index
LUQ: Left upper quadrant
RLQ: Right lower quadrant
MCR: Mean-crossing ratio
SBW: Spectral bandwidth
SC: Spectral centroid
MEMS: Micro-Electro-Mechanical systems
ANN: Artificial neural network
CNN: Convolutional neural network
SVM: Support vector machine
LSTM: Long short term memory
